# Prospective Evaluation of Changes in Pain Levels, Quality of Life and Functionality After Low Dose Radiotherapy for Epicondylitis, Plantar Fasciitis, and Finger Osteoarthritis

**DOI:** 10.3389/fmed.2020.00195

**Published:** 2020-05-19

**Authors:** Susanne Rogers, Brigitte Eberle, Deborah R. Vogt, Elisabeth Meier, Lorenz Moser, Silvia Gomez Ordoñez, Susanne Desborough, Oliver Riesterer, Istvan Takacs, Paul Hasler, Stephan Bodis

**Affiliations:** ^1^Center for Radiation Oncology KSA-KSB, Kantonsspital Aarau, Aarau, Switzerland; ^2^Clinical Trial Unit, Department of Clinical Research, University Hospital of Basel, University of Basel, Basel, Switzerland; ^3^Department of Physiotherapy, Kantonsspital Aarau, Aarau, Switzerland; ^4^Center for Radiation Oncology KSA-KSB, Kantonsspital Baden, Baden, Switzerland; ^5^Division of Rheumatology, Kantonsspital Aarau, Aarau, Switzerland; ^6^Department of Radiotherapy, University Hospital Zurich, Zurich, Switzerland

**Keywords:** radiotherapy, osteoarthritis, epicondylitis, plantar fasciitis, pain, quality of life, function, non-malignant

## Abstract

**Background:** The objective benefits of low dose radiotherapy (LDRT) for non-malignant joint disorders are controversial. This study evaluated changes in pain, quality of life (QoL) and function after LDRT for epicondylitis, plantar fasciitis, and finger osteoarthritis.

**Materials and Methods:** Patients over 40 years old with epicondylitis, plantar fasciitis, and finger osteoarthritis were had pain following at least 6 months of conservative therapy. Patients received 0.5 Gy LDRT twice weekly for 4 weeks repeated once after 8 weeks in patients who failed to achieve complete pain relief. Patients assessed their pain according to the visual analog scale. Handgrip strength was measured with an isometric dynamometer and the fast self-paced walking test was used in patients with plantar fasciitis. QoL was evaluated according to the EQ-5D and HAQ-DI questionnaires.

**Results:** Outcomes for 157 patients (204 sites) were documented at 2, 6, and 12 months after last LDRT. Pain reduction at rest (*p* < 0.001), during activity (*p* < 0.001) and increase in handgrip strength (extension *p* < 0.001, flexion *p* = 0.002) were highly significant for patients with lateral epicondylitis. Patients with medial epicondylitis reported pain relief at rest (*p* = 0.041) and during activity (*p* = 0.041) and significant increase in handgrip strength (*p* = 0.022). Patients with plantar fasciitis reported pain reduction at rest (*p* < 0.001), during activity (*p* < 0.001) and faster walking times (*p* < 0.001). A trend toward improved QoL was observed. Patients with finger osteoarthritis reported significant pain relief during activity (*p* < 0.001) and a gain in handgrip strength (*p* = 0.004), with a trend to both pain relief at rest (*p* = 0.056) and stronger pinch grip (*p* = 0.099).

**Conclusions:** LDRT achieved significant pain relief at rest and during activity and a corresponding objective improvement in handgrip strength in patients with epicondylitis. Pain relief at rest, during activity and improvement in walking time were demonstrated in patients with plantar fasciitis. LDRT achieved pain relief during activity, and handgrip strength was improved in patients with finger osteoarthritis. No significant effect was seen on quality of life measures for these conditions. The observed benefits were maintained 12 months after LDRT for all 3 indications and we recommend this low cost, safe intervention for patients over 40 who have failed prior conservative therapy.

## Background

Preclinical studies have shown that 0.3-1.0 Gy of radiation inhibits cell adhesion (1, 2) by increased production of TGF-beta1 (3, 4), reduced E-selectin expression in endothelial cells (1, 4) and induction of leucocyte apoptosis (5, 6). Radiation can also inhibit production of nitrous oxide in macrophages, leading to apoptosis (7) and reduced free radical production (8). These *in vitro* mechanisms have been substantiated in *in vivo* models (9, 10) and thus it is plausible that radiation-induced suppression of inflammatory cascades can achieve clinical pain relief in non-malignant musculoskeletal disorders with an active inflammatory component.

Arthritis and tendonitis are inflammatory conditions that arise in response to infectious, chemical or physical injury. They have different etiologies but common underlying inflammatory processes. Lateral and medial epicondylitis arise through repetitive strain injury that disrupts the connective tissue at the tendon insertion. The prevalence in adults over 40 years old is between 1 and 4% (11), particularly affecting the dominant arm (12–14). Plantar fasciitis affects 8–10% of the population with preponderance in females over the age of 40. Repeated microtrauma due to incorrect foot pronation (15), often exacerbated by a high body mass index and inappropriate footwear, can lead to chronic inflammation of the plantar fascia with pain on walking. Finger osteoarthritis affects 20–40% of the population aged 60–70 years of age in Europe and the United States, with a female to male ratio of 3:1. This degenerative joint disease is associated with low-grade inflammation that affects bone, ligaments, cartilage and synovial tissue. It is a heterogeneous condition with a multifactorial etiology and is one of the leading causes of disability worldwide (16).

The first line management of these inflammatory conditions may be pharmacological, typically topical or oral non-steroidal anti-inflammatory drugs, (NSAIDs) or non-pharmacological (17–19). The latter include instruction in joint protection techniques, assistive devices to help patients perform activities of daily living (ADLs), thermal modalities and splints (20). Low dose radiotherapy (LDRT) is reported to be an effective treatment for therapy-resistant non-malignant musculoskeletal disorders associated with only minimal toxicities and low costs (21).

Numerous retrospective series describe response rates of 63–89% following LDRT for non-malignant joint disorders (22). 7–70% of patients with epicondylitis benefited from a reduction in pain levels and complete pain relief was reported in 13–81% of patients following total radiation doses between 3.0 and 6.0 Gray (Gy), at 0.5–1.0 Gy per fraction and 2 fractions a week across several studies according to the von Pannewitz 4 point scale (23–25). A review of 2000 patients with epicondylitis treated with LDRT describes a response rate in excess of 70% in most publications. (23, 26–28). In the case of plantar fasciitis, 7–70% of patients reported some reduction in pain levels and complete pain relief was reported by 13–81% of patients (25). Randomized trials in patients with plantar fasciitis reported a superior effect with 6 Gy than 0.6 Gy suggesting a is consistent with a relevanffect of LDRT (29, 30), but no effect of fraction size could be seen (0.5 Gy vs. 1.0 Gy) (31). Over 75% of patients with osteoarthritis of the fingers and hand report pain relief, sustained for more than 5 years in 50%, even in those with a 10 year history of the condition (32) and more than 80% of patients benefit from a second course of LDRT (32).

In 2014 in Germany, 16,989 patients with non-malignant musculoskeletal conditions were irradiated to improve quality of life through pain relief and gain in function (33). Patients with non-malignant conditions account for 20% of the radiotherapy patient population in our center and low dose radiotherapy (LDRT) is frequently employed with good patient-reported outcomes. We therefore performed an evaluation of subjective changes in pain and quality of life and of objective changes in musculoskeletal function following low dose radiotherapy in patients with refractory pain who had failed standard pharmacological and non-pharmacological management options.

The aim of this study was to assess objective changes in musculoskeletal function in addition to patient-reported outcome measures (PROMs) in patients with lateral and medial epicondylitis, plantar fasciitis, and finger osteoarthritis following LDRT as applied in our practice.

## Methods

The trial was approved by the institutional review board (Ethikkommission Aargau-Solothurn, Aarau, Switzerland) and registered with the German Registry for Clinical Studies (No. DRK 500006288). Informed consent was obtained for inclusion of patients in the study. This prospective, uncontrolled interventional trial was performed in a single center setting and was conducted according to Good Clinical Practice and the declaration of Helsinki. The trial closed 12 months after the last patient was treated.

Consecutive patients with persistent pain from lateral and medial epicondylitis, plantar fasciitis and finger osteoarthritis who were referred for LDRT for were recruited between 01/2013 and 10/2015. Inclusion criteria were (1) age >40, (2) Karnofsky performance status >70, (3) non-pregnant or non-lactating if female, (4) effective contraception for 12 months if relevant and (5) prior treatment with conservative measures (physiotherapy, orthopedic splints or insoles and NSAIDs) for at least 6 months. Exclusion criteria were previous radiotherapy to the site, radiosensitivity disorders or an inability to comply with the study protocol.

**Figure 1:**
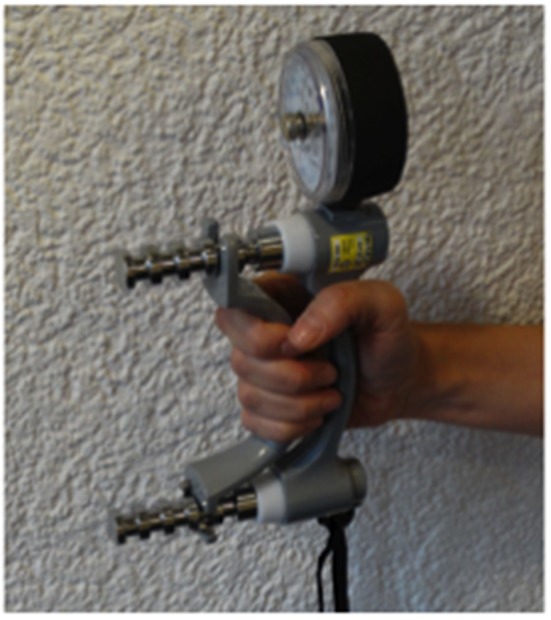
Isometric hydraulic dynanometer (JAMAR^®^, USA) for handgrip strength.

Informed consent was obtained by the radiation oncologist and patients completed two questionnaires validated in German: EuroQol-5 dimension (EQ-5D) for quality of life (34) and the Health Assessment Questionnaire (HAQ) (35, 36) for level of independence from which the Standard Disability Index was derived (DI). Patients scored their pain level between 0 and 10 according to the visual analog scale (VAS) at rest and during activity. A board-certified physiotherapist documented musculoskeletal function according to the anatomical site. Handgrip strength was tested in a sitting position using an isometric hydraulic dynamometer (JAMAR^®^, USA) (Figure 1) in elbow flexion for medial epicondylitis and in both flexion and extension for lateral epicondylitis.

**Figure 2:**
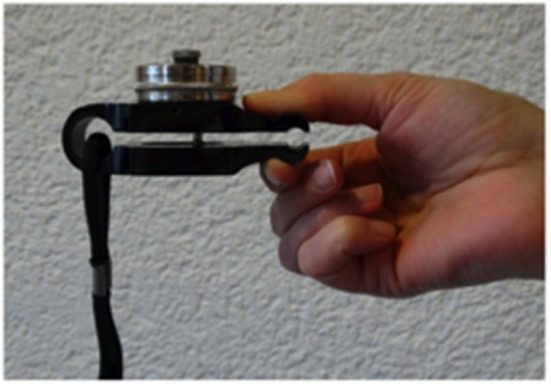
Isometric hydraulic dynanometer (JAMAR^®^, USA) for pinch grip strength.

Handgrip strength was tested in all patients with finger osteoarthritis, but pinch grip strength (37) only in those receiving thumb carpo-metacarpal irradiation using the designated JAMAR^®^ dynamometer (Figure 2).

Impairment of function due to plantar fasciitis was measured by the fast self-paced walking test (fSPWT), which counts the time taken to walk 20 meters (38). This simple test requires minimal instruction and has been used in randomized trials to measure the effect of therapeutic intervention due to robust reliability (37) and sensitivity to change in performance across diagnostic groups (39, 40).

Patients were irradiated with 8 x 0.5 Gy twice a week on non-consecutive days over 4 weeks to a total dose of 4.0 Gy with 200 kV orthovoltage direct fields (Therapax, Pantak, CT, USA). Non-target joints and, in the case of finger irradiation nail beds, were shielded with lead (Supplementary Figure 1). The dose prescription was the departmental standard at the time the study was initiated following reports of efficacy with a total dose between 3.0 and 6.0 Gy across the three indications (41).

LDRT was repeated up to a dose of 8.0 Gy in patients who did not achieve a complete pain response with the first course (42). Follow-up tests of functionality (handgrip, pinch grip strength and fast self-paced walking test) were performed at 2, 6, and 12 months after irradiation. VAS pain scores, and EQ-5D and HAQ-DI questionnaires were completed by the patients at these visits.

### Statistical Analysis

The study endpoints were VAS pain scores, handgrip and pinch grip strength (kg), walking time (seconds), EQ-5D score (calculated on the regression coefficients of the lean model for the German reference population) and HAQ-DI score. Outcomes were evaluated per site and patients with lateral epicondylitis, medial epicondylitis, finger osteoarthritis and plantar fasciitis were analyzed separately.

Descriptive summary statistics (mean, standard deviation, median, interquartile range, minimum and maximum, number of available data) are presented for each time point for each analysis set (Supplementary Tables 1–4). The differences compared with baseline at 2, 6, and 12 months after LDRT appeared so large that *post hoc* testing of median changes was performed (paired Wilcoxon signed rank test). The two-sided *p*-values were adjusted for multiple comparisons (Bonferroni-Holm method). An adjusted *p*-value of less than 0.05 was considered statistically significant. Spearman's rank correlation coefficient (rho) was calculated to assess the relationship between changes in pain scores and functional test outcomes for lateral epicondylitis and plantar fasciitis. A smoothing function was fitted to the EQ-5D and the HAQ-DI scores using locally weighted smoothing (LOESS) to allow an estimation of the overall time trend. All statistical analyses were conducted using the statistical software package R (43).

## Results

One hundred and fifty-seven patients [lateral epicondylitis (35), medial epicondylitis (9), finger osteoarthritis (59) and plantar fasciitis (54)] with 204 sites were treated: lateral epicondylitis (39), medial epicondylitis (10), finger osteoarthritis (99) and plantar fasciitis (56). The low number of patients with medial epicondylitis reflects its incidence. All patients reported limitations in activities of daily living (ADL) due to pain at rest or during activity. Patient characteristics are summarized in Table 1.

**Table 1:** Patient characteristics for each analysis set.

	**Lateral epicondylitis**	**Medial epicondylitis**	**Finger osteoarthritis**	**Plantar fasciitis**
No. of patients	35	9	59	54
Age	50.0 (7.8)	56.8 (6.5)	62.4 (9.0)	52.4 (7.7)
Gender = female	20 (57.1)	7 (77.8)	52 (88.1)	43 (79.6)
Total.sessions = 2	22 (62.9)	7 (77.8)	46 (78.0)	34 (63.0)
Irradiated side				
Bilateral	4 (11.4)	1 (11.1)	40 (67.8)	2 (3.7)
Left	11 (31.4)	4 (44.4)	10 (16.9)	22 (40.7)
Right	20 (57.1)	4 (44.4)	9 (15.3)	30 (55.6)

*Age: mean (s.d.), other variables: frequency (%)*.

Complete pain response (VAS 0) was achieved in 55/204 (27%) of sites after the first course of low dose radiotherapy and therefore the second course of LDRT was not given in these patients. The planned second course of LDRT was delivered in 149/204 (73%) sites after 2–12 months (25/39 lateral epicondylitis, 8/10 medial epicondylitis, 81/99 finger osteoarthritis, 35/56 plantar fasciitis). Eighty percent (165/204) of treated sites could be assessed at 12 months (30/38 lateral epicondylitis, 6/10 medial epicondylitis, 82/100 finger osteoarthritis for handgrip and 53/65 for pinch grip, 30/38 plantar fasciitis). All patients who attended for follow-up at 2, 6, and 12 months returned the quality of life questionnaires.

The data points for each set at 2, 6, and 12 months after last LDRT (12 months after the first course if one course was given or the second course in the event of 2 courses of irradiation) are shown in violin plots (Supplementary Figures 2–5). The median changes in pain and outcome scores 2, 6, and 12 months after last LDRT compared with baseline are summarized in Table 2.

**Table 2:** Median changes and p-values from the Wilcoxon signed rank test for different outcomes of each analysis set assessed at 2, 6, and 12 months after the last LDRT session compared to the baseline values before the start of the first LDRT session.

**Data set**	**Functional test**	**Time after LDRT (months)**	**Median change [IQR]**	***p*–value**
Lateral epicondylitis	Pain at rest	2	−2 [−5, 0]	<0.001
	Pain at rest	6	−1 [−4.25, 0]	0.01
	Pain at rest	12	−2.5 [−5, 0]	<0.001
	Pain during activity	2	−4 [−6, −2]	<0.001
	Pain during activity	6	−5 [−7, −2]	<0.001
	Pain during activity	12	−6 [−7, −4]	<0.001
	Handgrip in flexion	2	4 [−2, 9]	0.016
	Handgrip in flexion	6	4.5 [0, 15]	0.002
	Handgrip in flexion	12	5.25 [0, 11.75]	0.002
	Handgrip in extension	2	9.5 [−0.075, 20.75]	<0.001
	Handgrip in extension	6	16 [5, 23]	<0.001
	Handgrip in extension	12	16 [5.5, 23.75]	<0.001
Medial epicondylitis	Pain at rest	2	−2.5 [−3.375, −0.75]	0.04
	Pain at rest	6	−3 [−5.75, −2.5]	0.04
	Pain at rest	12	−3 [−4.5, −2.25]	0.04
	Pain during activity	2	−2.5 [−4.25, −0.75]	0.04
	Pain during activity	6	−4 [−6.25, −3]	0.045
	Pain during activity	12	−4 [−6.25, −3.25]	0.04
	Handgrip in flexion	2	−4 [−5, 3]	0.722
	Handgrip in flexion	6	6 [4, 7.25]	0.04
	Handgrip in flexion	12	6.5 [4.25, 8.5]	0.022
Finger osteoarthritis	Pain at rest	2	−0.75 [−3, 0]	<0.001
	Pain at rest	6	−1 [−4, 0]	<0.001
	Pain at rest	12	0 [−3.5, 0.25]	0.03
	Pain during activity	2	−1.5 [−4, 0]	<0.001
	Pain during activity	6	−2.25 [−5, 0]	<0.001
	Pain during activity	12	−3 [−5, 0.5]	<0.001
	Handgrip in flexion	2	2 [−2, 5.5]	0.026
	Handgrip in flexion	6	3 [−3, 7]	0.016
	Handgrip in flexion	12	2.5 [−1, 6]	0.004
	Pinch grip	2	0.5 [−0.5, 1.5]	0.054
	Pinch grip	6	0.5 [−0.125, 2]	0.001
	Pinch grip	12	0.5 [−0.5, 1.625]	0.101
Plantar fasciitis	Pain at rest	2	−3 [−5, 0]	<0.001
	Pain at rest	6	−4 [−6, 0]	<0.001
	Pain at rest	12	−4 [−6, 0]	<0.001
	Pain during activity	2	−4 [−6.25, −2.75]	<0.001
	Pain during activity	6	−6.5 [−8, −3.75]	<0.001
	Pain during activity	12	−6 [−8, −4]	<0.001
	Walking test	2	−2 [−6, −0.25]	<0.001
	Walking test	6	−4 [−6, −1.5]	<0.001
	Walking test	12	−5 [−8, −2.5]	<0.001

*P-values were adjusted for multiple comparisons using the Bonferroni-Holm method. Pain was scored with the visual analog scale (VAS). Median handgrip and pinch grip strength are reported in kilograms and walking time is in seconds*.

### Lateral Epicondylitis

Patients with lateral epicondylitis had higher baseline median pain scores during activity than at rest (6.0 vs. 3.0). A median pain score of 0 at rest was reached 2 months after LDRT (*p* = 0.006), and during activity after 12 months (*p* = 0.002). This pattern was observed after the first and second course of LDRT. Median change in pain score 12 months after last LDRT was −2.5 (*p* < 0.001) at rest, and −6.0 (*p* < 0.001) during activity. At rest, 23/31 (74%) treated sites responded completely and 21/27 (78%) patients were pain free at 12 months. During activity, 13/30 (43%) treated sites and 11/28 (39%) patients were pain free at 12 months. Median change in handgrip strength 12 months after last LDRT was 5.2 kg (*p* = 0.002) in elbow flexion and 16.0 kg (*p* < 0.001) in elbow extension (Table 2). There were inverse correlations between handgrip strength in elbow extension and pain at rest (rho = −0.6, *p* < 0.001) and pain during activity (rho = −0.5, *p* < 0.001) (Table 3). No trend was visible for the EQ-5D total score for patients with lateral epicondylitis whilst for the HAQ-DI, there may have been a slight decrease over time (Supplementary Figure 6).

**Table 3:** Correlations between changes in pain (VAS) and functional outcomes of specific interest after last LDRT session.

**Data set**	**Change between**	**Correlation**	**Spearman's rho [95% CI]**	***p*–value**
Lateral epicondylitis	0–2 months	Handgrip flexion vs. pain at rest	−0.5 [−0.71, −0.21]	0.009
	0–2 months	Handgrip flexion vs. pain during activity	−0.64 [−0.8, −0.4]	<0.001
	0–6 months	Handgrip flexion vs. pain at rest	−0.3 [−0.59, 0.05]	0.138
	0–6 months	Handgrip flexion vs. pain during activity	−0.03 [−0.38, 0.32]	0.842
	0–12 months	Handgrip flexion vs. pain at rest	−0.54 [−0.76, −0.22]	0.01
	0–12 months	Handgrip flexion vs. pain during activity	−0.39 [−0.66, −0.02]	0.086
	0–2 months	Handgrip extension vs. pain at rest	−0.46 [−0.68, −0.16]	0.011
	0–2 months	Handgrip extension vs. pain during activity	−0.7 [−0.84, −0.49]	<0.001
	0–6 months	Handgrip extension vs. pain at rest	−0.38 [−0.64, −0.03]	0.054
	0–6 months	Handgrip extension vs. pain during activity	−0.33 [−0.61, 0.02]	0.333
	0–12 months	Handgrip extension vs. pain at rest	−0.47 [−0.71, −0.13]	0.02
	0–12 months	Handgrip extension vs. pain during activity	−0.36 [−0.64, 0.02]	0.092
Medial epicondylitis	0–2 months	Handgrip flexion vs. pain at rest	−0.35 [−0.85, 0.47]	0.649
	0–2 months	Handgrip flexion vs. pain during activity	−0.35 [−0.85, 0.47]	0.239
	0–6 months	Handgrip flexion vs. pain at rest	0.15 [−0.68, 0.81]	0.607
	0–6 months	Handgrip flexion vs. pain during activity	−0.65 [−0.94, 0.21]	0.037
	0–12 months	Handgrip flexion vs. pain at rest	−0.32 [−0.9, 0.66]	0.392
	0–12 months	Handgrip flexion vs. pain during activity	−0.55 [−0.94, 0.47]	0.58
Finger osteoarthritis	0–2 months	Handgrip vs. pain at rest	−0.28 [−0.46, −0.09]	0.031
	0–2 months	Handgrip vs. pain during activity	−0.36 [−0.52, −0.18]	0.011
	0–6 months	Handgrip vs. pain at rest	−0.24 [−0.43, −0.03]	0.049
	0–6 months	Handgrip vs. pain during activity	−0.42 [−0.58, −0.22]	0.001
	0–12 months	Handgrip vs. pain at rest	−0.35 [−0.53, −0.14]	0.002
	0–12 months	Handgrip vs. pain during activity	−0.48 [−0.63, −0.28]	<0.001
	0–2 months	Pinch grip vs. pain at rest	−0.3 [−0.51, −0.05]	0.032
	0–2 months	Pinch grip vs. pain during activity	−0.36 [−0.56, −0.12]	0.01
	0–6 months	Pinch grip vs. pain at rest	−0.32 [−0.54, −0.05]	0.031
	0–6 months	Pinch grip vs. pain during activity	−0.31 [−0.54, −0.04]	0.036
	0–12 months	Pinch grip vs. pain at rest	−0.31 [−0.54, −0.03]	0.054
	0–12 months	Pinch grip vs. pain during activity	−0.29 [−0.52, −0.01]	0.1
Plantar fasciitis	0–2 months	Walking test vs. pain at rest	0.36 [0.09, 0.58]	0.054
	0–2 months	Walking test vs. pain during activity	0.49 [0.25, 0.68]	0.012
	0–6 months	Walking test vs. pain at rest	0.4 [0.12, 0.63]	0.011
	0–6 months	Walking test vs. pain during activity	0.41 [0.13, 0.63]	0.009
	0–12 months	Walking test vs. pain at rest	0.31 [−0.01, 0.58]	0.239
	0–12 months	Walking test vs. pain during activity	0.43 [0.13, 0.66]	0.049

*P-values were adjusted for multiple comparisons using the Bonferroni-Holm method. Rho=Spearman's rank correlation coefficient*.

### Medial Epicondylitis

12 months after last LDRT, median pain scores at rest and during activity were both 0 (*p* = 0.331 and *p* = 0.249, respectively). At rest, 5/6 treated sites (83%) were pain free at 12 months. Median changes in pain 12 months after last LDRT were: −3.0 (*p* = 0.041) at rest and −4.0 (*p* = 0.041) during activity (Table 2). Median change in handgrip strength after 12 months showed a significant increase (6.5 kg, *p* = 0.022). Quality of life seemed to increase after radiotherapy according to the EQ-5D but not the HAQ-DI score (Supplementary Figure 7).

### Finger Osteoarthritis

Pain at rest due to finger osteoarthritis was low prior to therapy (median: 2.0) and did not decrease significantly after last LDRT (median change = 0, *p* = 0.056). At rest, 40/83 (48%) treated sites (27/48: 56% patients) were pain free at 12 months. In contrast, pain during activity was high at baseline (median: 5.0) and decreased significantly after last LDRT (median change = −3.0, *p* < 0.001). During activity, 11/83 (13%) treated sites (9/48: 19% patients) had VAS scores of 0 at the same time point. A significant improvement was seen for handgrip strength (median change = 2.5 kg, *p* = 0.004) but not pinch grip (median change 0.5 kg, *p* = 0.099) at 12 months. No clear trend was visible in either EQ-5D total score or HAQ-DI (Supplementary Figure 8).

### Plantar Fasciitis

Both pain at rest and during activity decreased rapidly after radiotherapy for plantar fasciitis. Pain at rest decreased from a median VAS of 1.0–0.0 2 months after last LDRT (median change = 0.0, p = 0.003) and was sustained for 12 months. Median change in pain at rest at 12 months was −4.0 (*p* < 0.001). 38 of 41 (93%) sites evaluated at 12 months (38/41 patients, 93%) were pain free. In comparison, pain scores during activity were higher before LDRT (VAS: 6.0) but decreased to a VAS of 0 after 6 months (median change = −4.0, *p* < 0.001). 28 of 41 sites evaluated at 12 months (28/41 patients, 68%) were pain free during activity. Median change in pain during activity 12 months after last LDRT was −6.0 (*p* < 0.001). Performance in the fSPWT also improved significantly. At 12 months after LDRT, the median walking time was 5.0 s quicker (*p* < 0.001) compared with before first LDRT. At 12 months, the correlation between walking time and pain during activity (rho = 0.5, *p* < 0.001) was stronger than with pain at rest (rho = 0.2, *p* = 0.008). There seemed to be a slight increase in EQ-5D total score) but not for the HAQ-DI (Supplementary Figure 9).

## Discussion

This study demonstrates effective pain relief and objective gain in musculoskeletal function with LDRT across multiple non-malignant joint disorders.

Other than a dose finding study (26), this is the first prospective account of the pain response to LDRT in patients with lateral epicondylitis and medial epicondylitis and the first to document a significant parallel improvement in handgrip strength. Together with a retrospective review comprising 2,141 patients with epicondylitis (23), this cohort confirms the ability of LDRT to achieve 63–83% complete pain control in this condition. Due to the low number of patients with medial epicondylitis (*n* = 9), the data cannot be considered statistically meaningful, however the median 6.5 kg increase in handgrip strength 12 months after LDRT and pain reduction of 3–4 VAS points strongly suggest efficacy in both medial epicondylitis and lateral epicondylitis.

Patients with finger osteoarthritis and pain during activity reported pain relief following LDRT, however, the reduction in pain relief at rest did not quite reach statistical significance, although there was a trend to improvement 12 months after LDRT as compared with prior to first LDRT. The low baseline VAS scores at rest-16/59 patients (27%) had median VAS 0 at rest but pain during activity-may have been a contributory factor. This pattern of pain during activity but not at rest is characteristic of osteoarthritis (44).

This study confirms the effectiveness of LDRT for the pain relief of plantar fasciitis, consistent with complete response rates of up to 81% reported in a review of 11,909 patients from 22 studies (22). Predictive factors for response to LDRT for plantar fasciitis include a megavoltage technique and one treatment series (45). We used orthovoltage radiotherapy but obtained response rates comparable to those achieved with megavoltage irradiation, therefore the 200 kV beam profile was adequate. Two courses of LDRT were given in 34/54 (63%) patients with plantar fasciitis who were not pain free 2–12 months after the first course of radiotherapy, but sustained pain control at 12 months was also feasible with one course of total 4.0 Gy in 20 (37%) patients.

We showed a corresponding improvement in function after LDRT for plantar fasciitis. Guidelines from the American Physical Therapy Society recommend: “… easily reproducible activity limitation and participation restriction measures associated with their patient's heel pain/plantar fasciitis to assess the changes in the patient's level of function…” (46). The fSPWT is a reliable performance indicator in patients with osteoarthritis of the hip and knee (47, 48), although re-testing within 21 days has shown some variability (49). We were unable to control for normal variability in our cohort, however median walking times were 5 s faster (23 times the variability reported above) which indicates a genuine effect. The positive correlation between reduction in pain levels during activity and walking times suggests this test could become a validated functional test for plantar fasciitis.

The improved pain and function test scores did not translate into significant improvements in quality of life or improvement in functional independence for any subgroup as evaluated by EQ-5D and HAQ-DI, respectively. Trends to increased function and decreased invalidity could be seen in 50% of the analysis sets (EQ-5D: medial and lateral epicondylitis, HAQ-DI: medial epicondylitis and plantar fasciitis), however the medial epicondylitis group contained only a small number of patients. It is possible that these questionnaires did not clearly show a corresponding benefit in quality of life as the questions address practical issues which are likely to be limited by chronic joint deformity (50) on which LDRT has no impact. The EQ-5D is a validated but generic questionnaire and it has been suggested that the combination of both a generic and a disease-specific quality of life measure (51) can better detect treatment effects (52). A recent evaluation of the HAQ-DI in generalized osteoarthritis found good construct validity, internal consistency and reliability but that its content validity and responsiveness were limited and adaptations were recommended (53). Furthermore, the EQ-5D has been mainly utilized in hip and knee osteoarthritis rather than the conditions studied in this trial, which may explain why little benefit on quality of life was documented despite a substantial effect on pain.

The main limitation of this study is that concomitant analgesia could not be interpreted as non-malignant musculoskeletal conditions such as osteoarthritis usually affect more than one anatomical site and dosage may be altered due to pain at a non-irradiated site. Although a transient pain flare may require additional medication, the subsequent sustained analgesic benefits of radiotherapy can reduce the need for long-term analgesia and the associated potential toxicities (54). Study patients had failed to respond to at least 6 months of conservative therapies including physiotherapy, non-steroidal anti-inflammatory analgesia and orthopedic splints or insoles and it is very unlikely that the reduction in pain and gain in function were due to spontaneous improvement.

Randomized data for LDRT in non-malignant conditions are scarce (30, 31, 55, 56) and without adequately powered randomized controlled trials (RCT), the benefit of LDRT cannot be concluded. A previous clinical trial with 62 patients with plantar fasciitis showed superior efficacy of 6.0 Gy over 0.6 Gy and was closed early on ethical grounds, strongly supporting a real benefit from radiotherapy (29). The statistical correlations between pain relief and functional outcomes across the disease entities in the current study are consistent with a relevant effect of LDRT. Although the study was uncontrolled, the lack of blinding is not expected to have had any significant impact on the objective functional tests (57).

Two recent sham-controlled RCTs failed to demonstrate any beneficial effect of 1.0 Gy weekly for 6 weeks in patients with osteoarthritis of the hand (56) and knee (55). Sham irradiation raises ethical questions however because the radiotherapy team must engage in active deception as only the patient is unaware whether radiotherapy has been delivered or not. Of note, neither of the above trials included objective functional tests as utilized in this study. The strength of handgrip and pinch grip have been found to be reliable and sensitive to change in patients with osteoarthritis of the hand (58). The association between grip strength and questionnaires assessing physical function is reportedly limited and the latest recommendations of the Osteoarthritis Research Society (OARSI) are to include grip strength as an outcome to improve the quality of clinical trials (59), as performed in this study. We found the changes in musculoskeletal function could be meaningfully evaluated using the handgrip strength dynamometer and the fast self-paced walking test may be usefully incorporated into future clinical trials non-malignant disorders of the lower limb.

The primary endpoint of the above sham-controlled RCT was “proportion of responders” (i.e., pain relief and subjective assessment of function) and this was not statistically different in the experimental (29% hand, 44% knee) and the sham (36% hand, 43% knee) groups. Patients received only one course of LDRT and the primary endpoint was assessed after only 3 months. In our study, two courses of LDRT were standard and received by 73% of patients who had an incomplete pain response after the first course. It has been observed that patients with plantar fasciitis, epicondylitis, and hand osteoarthritis benefit from a second course of LDRT after 8 weeks (60). A large retrospective cohort of patients with arthritis and enthesopathies reported that response to therapy developed many months after completion of therapy: a median VAS score of 7.0 prior to LDRT, 5.0 immediately after, 4.0 after 12 weeks and 1.0 after 38 months (61). In contrast, longer follow-up of the patients with hand osteoarthritis in the sham-controlled randomized trial of LDRT with a single course of 6 × 1.0 Gy weekly showed no difference in benefit between the experimental and sham groups at 6 and at 12 months (62). A further randomized trial is needed to ascertain whether the apparent benefit in the previous studies is due to regression to the mean or placebo effect or indeed LDRT. The data from the Arthorad randomized trial (6 × 0.5 Gy vs. 6 × 0.05Gy) from the DEGRO group are awaited at the end of 2020. We therefore endorse the comments by Ott et al. (63) that these trials are not yet practice changing as patient numbers were low, the prescription of 1.0 Gy weekly for 6 weeks was not standard, and that the 3 month follow-up is short. We also report lesser clinical benefit of LDRT in patients with finger osteoarthritis, which we attribute to underlying complex joint deformities that are not expected to respond to LDRT. The benefits of LDRT for patients with pain at rest from finger osteoarthritis and could not be demonstrated in our study, putatively due to low baseline pain levels. Osteoarthritis data should not be extrapolated to epicondylitis and plantar fasciitis where LDRT is highly successful. Our study protocol used the 2013 departmental standard of 8 × 0.5 Gy, however our current practice is 6 × 0.5 Gy (31) repeated after a minimum interval of 8 weeks in the absence of a complete pain response.

Many countries do not offer radiotherapy for benign conditions, mostly due to concerns about the potential for radiation-induced malignancy. As the population in many countries is aging and such disorders result in significant impairment in quality of life and costs, there is now a wider international interest in LDRT (64). As a precaution, we do not offer LDRT to patients under 40 years old or to those whose family planning is not yet complete, although dose to the reproductive organs is low (32). No patients in our cohort reported any toxicity. Acute skin toxicities greater than grade 1 are rare (23, 29) and, importantly, no induced tumors have ever been reported (30, 65). As the median age of LDRT patients is 65 years, any potential risks should be offset by the potential benefits (66).

## Conclusions

Patients with refractory pain from lateral epicondylitis, plantar fasciitis and finger osteoarthritis with pain during activity reported significant pain relief and demonstrated improved musculoskeletal function sustained after 12 months. The improved pain and functional test scores were not associated with improved quality of life as evaluated by the EQ-5D and HAQ-DI questionnaires. LDRT with 6 x 0.5 Gy, repeated after a minimum of 8 weeks in the absence of a complete pain response, may be offered to patients over 40 years old as a safe, non-toxic treatment modality which may achieve sustained pain relief and improved functional outcomes 12 months after the last treatment.

## Data Availability Statement

The datasets used and/or analyzed during the current study are available from the corresponding author on reasonable request.

## Ethics Statement

The studies involving human participants were reviewed and approved by Aargau Ethikkommission (2012/069). The patients/participants provided their written informed consent to participate in this study. Informed consent to participate was obtained for all patients.

## Author Contributions

BE, IT, EM, PH, and SB designed the trial. DV performed the statistical analysis. SR wrote the manuscript. OR and SB reviewed the manuscript. BE, EM, SD, SG, and LM collected data. All authors approved the final manuscript.

## Conflict of Interest

The authors declare that the research was conducted in the absence of any commercial or financial relationships that could be construed as a potential conflict of interest.

## Abbreviations

PROMs: patient-reported outcome measures
LDRT: low dose radiotherapy
VAS: visual analog scale
QoL: quality of life
fSPWT: fast self-paced walking test
HAQ-DI: Health Assessment Questionnaire-disability index
EQ-5D: EurolQol-5 dimensions
DRK: Deutsches Register Klinische Studien.
