# Supplementary material for: Prospective Evaluation of Changes in Pain Levels, Quality of Life and Functionality After Low Dose Radiotherapy for Epicondylitis, Plantar Fasciitis, and Finger Osteoarthritis
Source: Front Med (Lausanne). 2020 May 19;7:195. doi: 10.3389/fmed.2020.00195 (PMC7249275; doi:10.3389/fmed.2020.00195)
Supplement: Supplementary file 1 [file Data_Sheet_1.docx]

**Supplementary Figures**

**Supplementary Figure 1**: Simulated patient set -up for finger osteoarthritis irradiation with nailbed and non-target joint shielding on an orthovoltage unit.

**
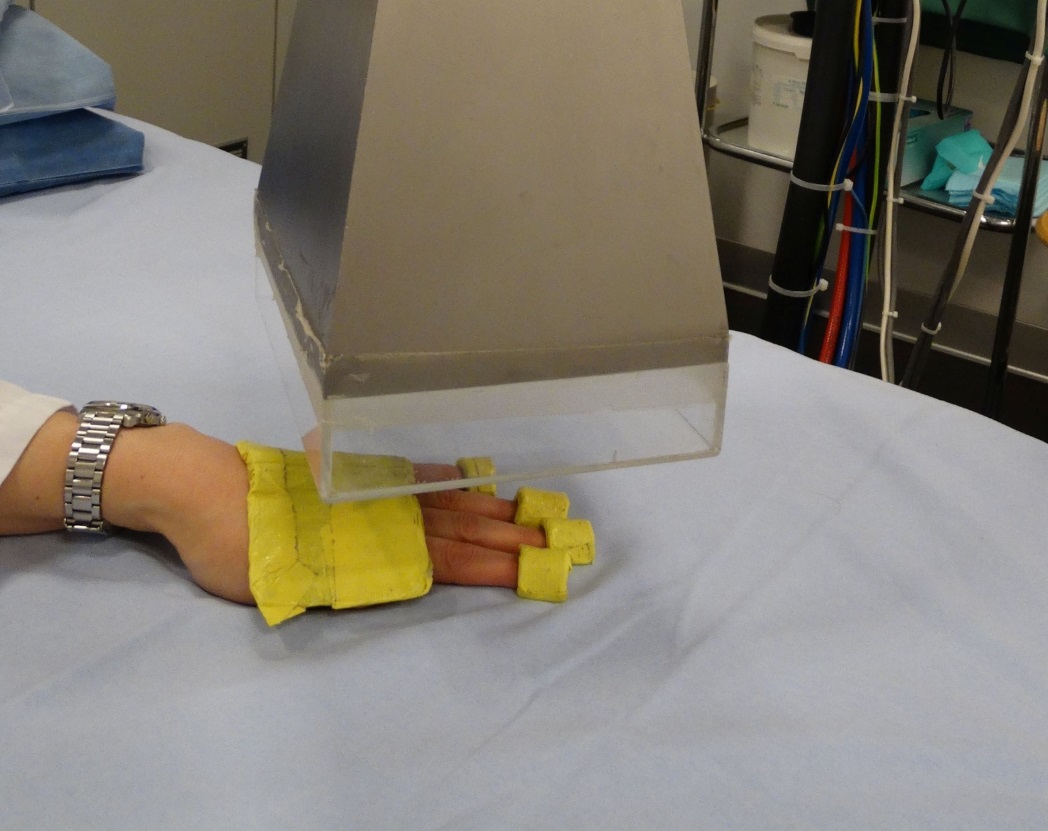
**

**Supplementary Figure 1**

**Supplementary Figures 2, 3, 4 and 5.**

The colored ‘violins’ represent the density distributions of the outcome variable. The ‘whiskers’ represent observations within 1.5 times the interquartile range above and below the median (red dot).

**Supplementary Figure 2**.

Lateral epicondylitis outcomes after the last course of radiotherapy (either first or second) compared with corresponding baseline values. (2a) Pain at rest (VAS), (2b) pain during activity (VAS), (2c) handgrip strength in elbow flexion, (2d) handgrip strength in elbow extension (kg).

**Supplementary Figure 2**


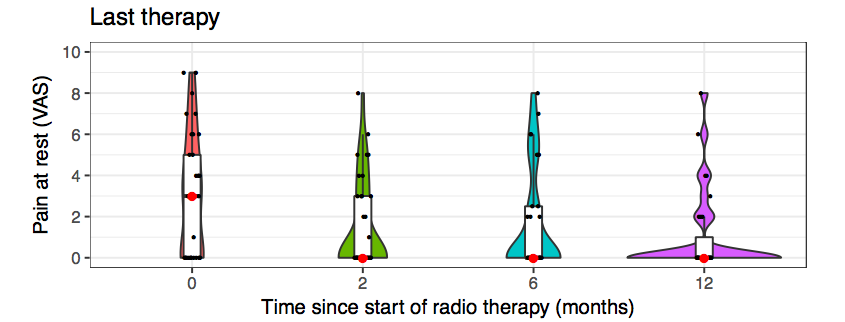


Figure 2a


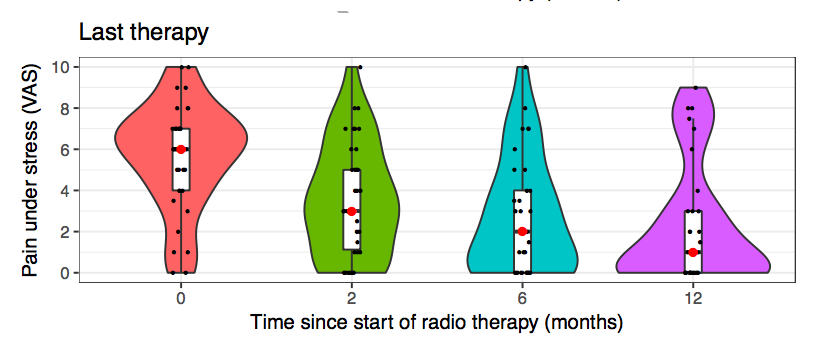


Figure 2b


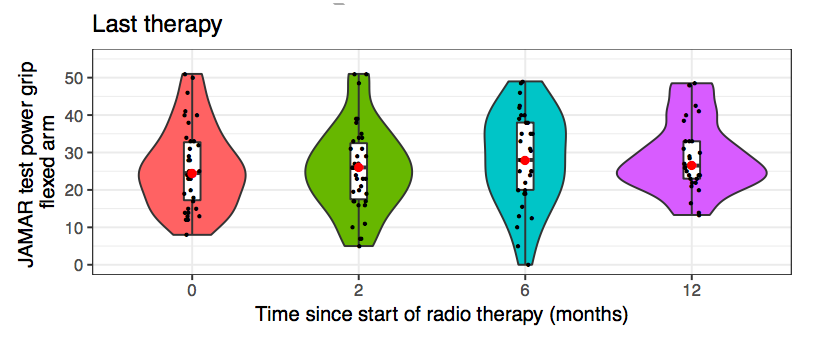


Figure 2c


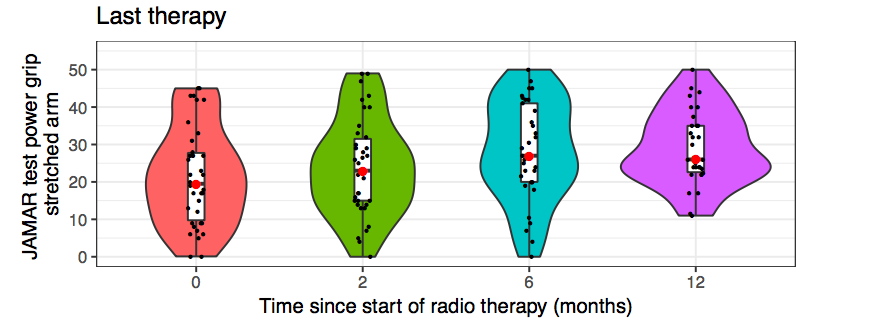


Figure 2d

**Supplementary Figure 3.**

Medial epicondylitis outcomes after the last course of radiotherapy (either first or second) compared with corresponding baseline values: (3a) Pain at rest (VAS), (3b) pain during activity (VAS), (3c) handgrip strength in elbow flexion (kg).

**Supplementary Figure 3**


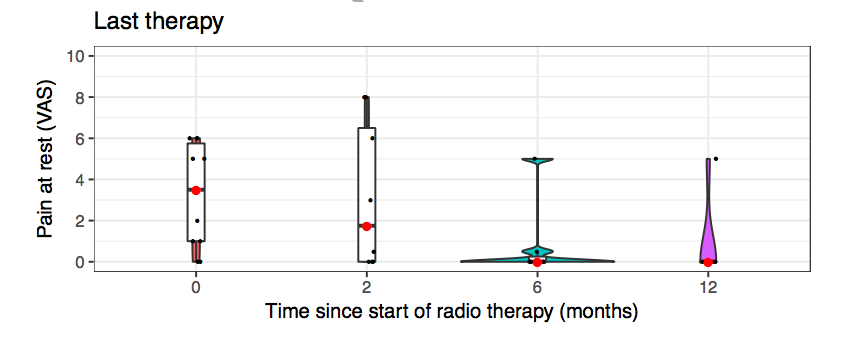


Figure 3a


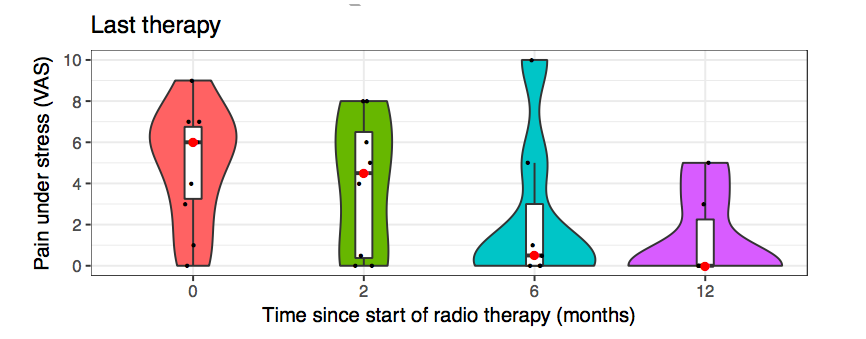


Figure 3b


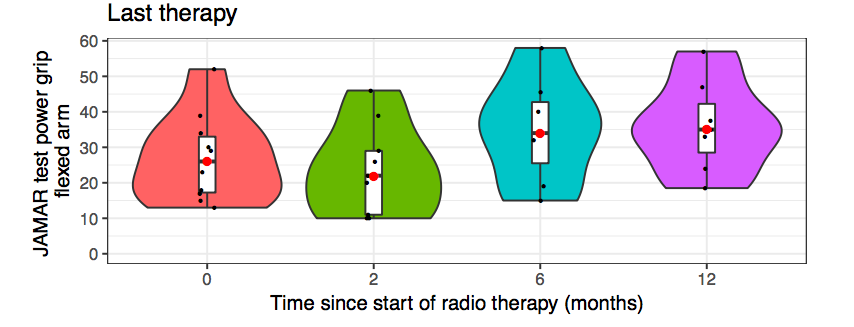


Figure 3c

**Supplementary Figure 4.**

Plantar fasciitis outcomes after the last course of radiotherapy (either first or second) compared with corresponding baseline values. (4a) Pain at rest (VAS), (4b) pain during activity (VAS), (4c) walking test (seconds).

**Supplementary Figure 4**


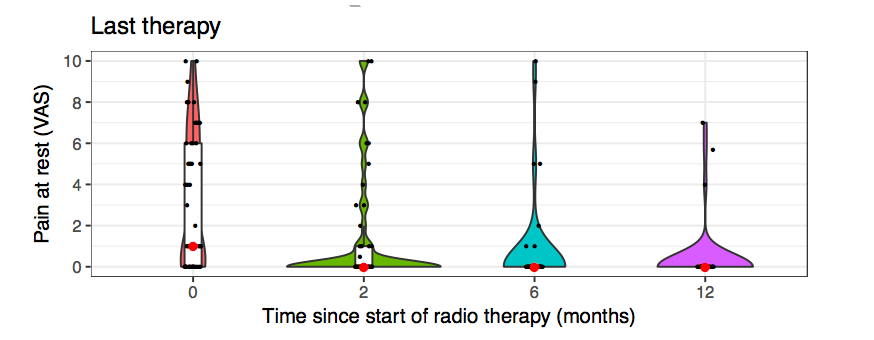


Figure 4a


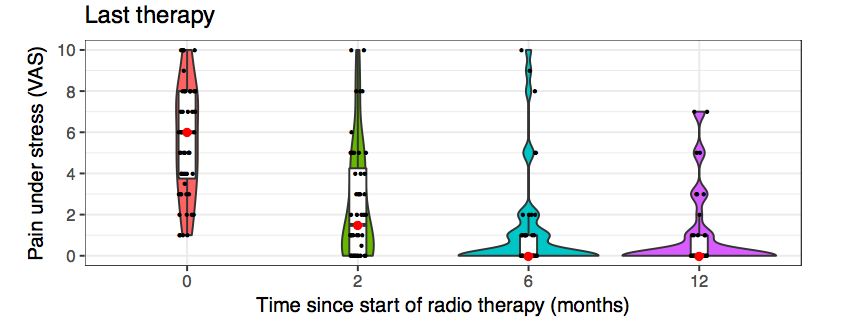


Figure 4b


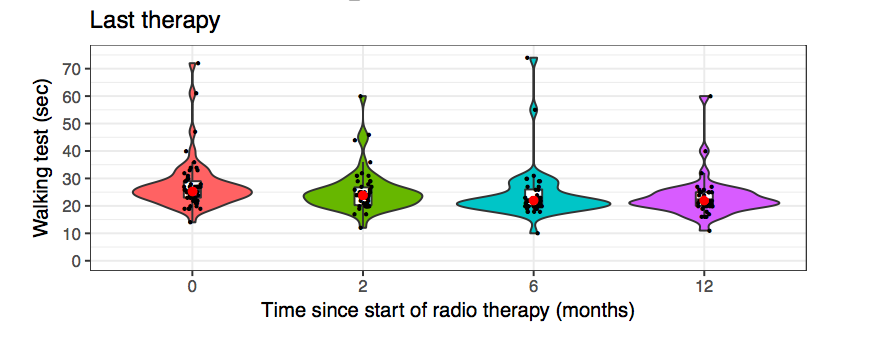


Figure 4c

**Supplementary Figure 5**.

Finger osteoarthritis after last course of radiotherapy (either first or second) compared with corresponding baseline values. (5a) Pain at rest (VAS), (5b) pain during activity (VAS), (5c) handgrip strength, (5d) pinch grip strength.

**Supplementary Figure 5**


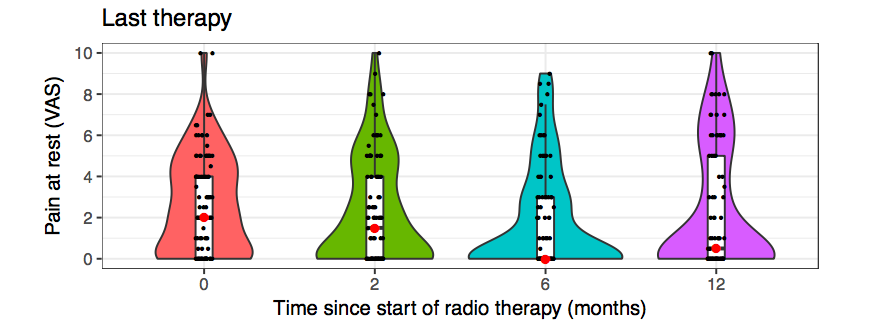


Figure 5a


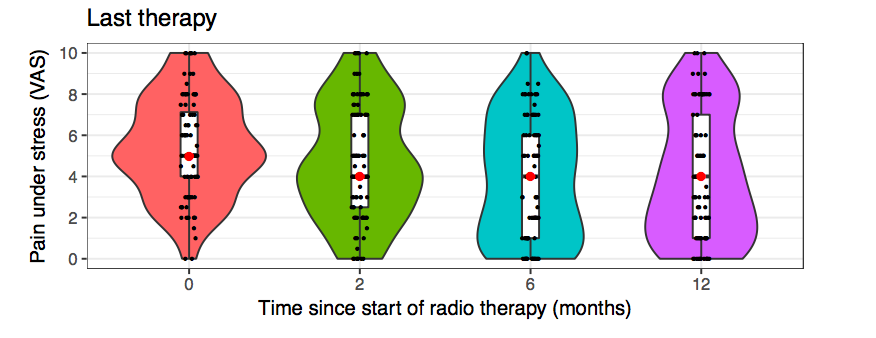


Figure 5b


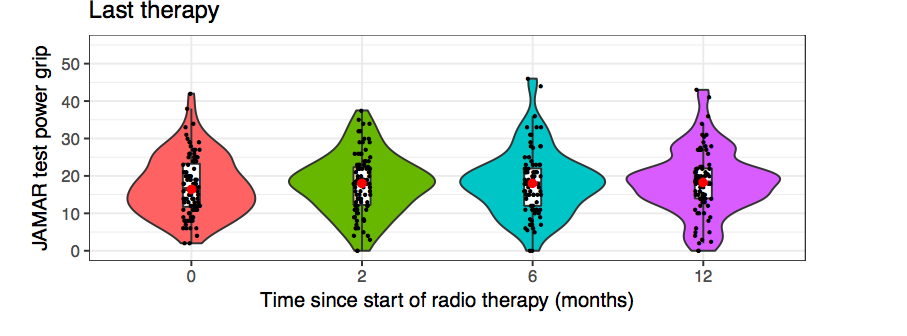


Figure 5c


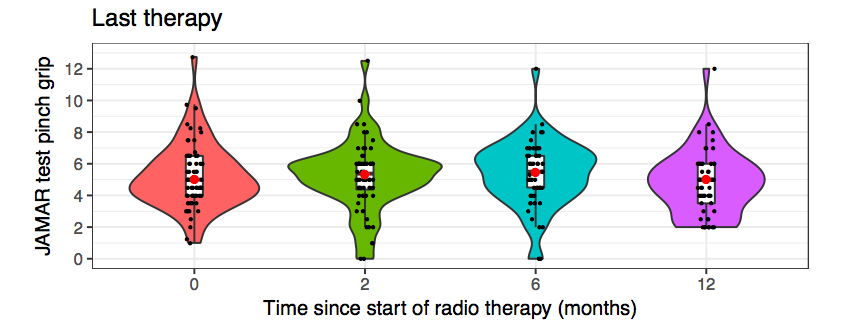


Figure 5d

**Supplementary Figure 6.**

Lateral Epicondylitis: No trend is visible for EQ-5D total score, while for HAQ DI, there might be a slight decrease with time.


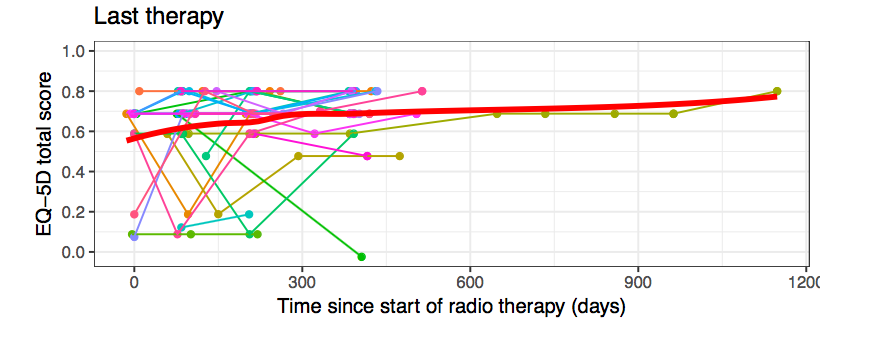


Supplementary Figure 6a


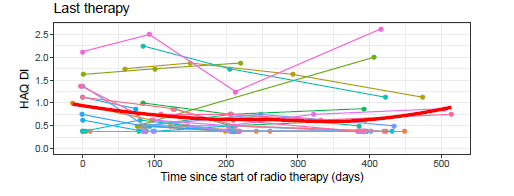


Supplementary Figure 6b

**Supplementary Figure 7.**

Medial epicondylitis: Quality of life seems to increase after radiotherapy. There is an increase in EQ-5D total score and a decrease in HAQ DI.

Supplementary Figure 7


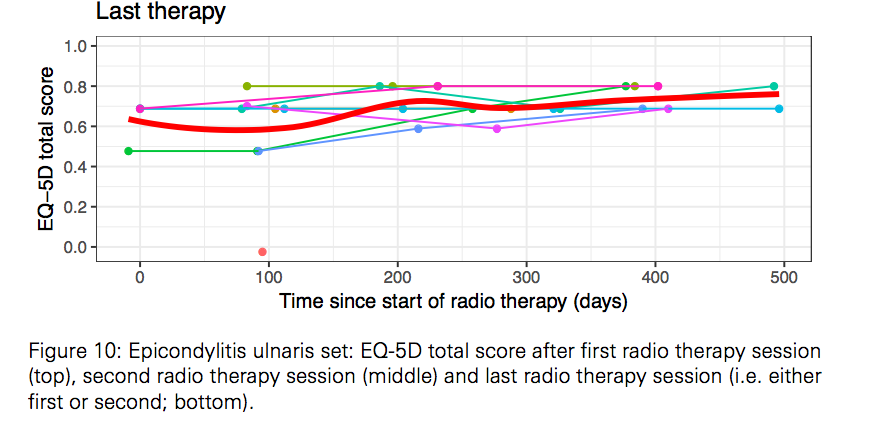


Supplementary Figure 7a


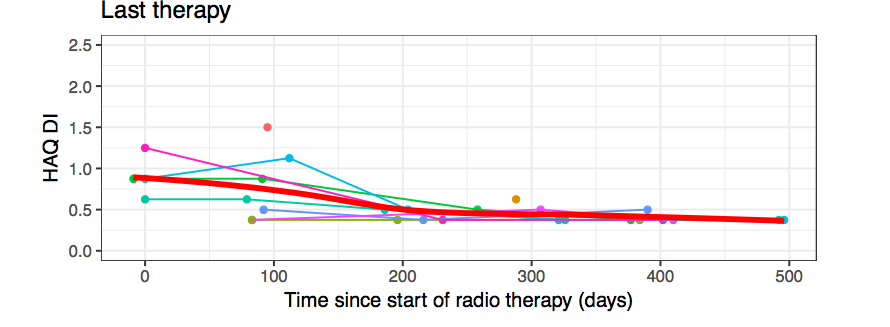


Supplementary Figure 7b

Supplementary Figure 8.

Finger osteoarthritis: No clear trend was visible in either ED-5D total score of HAQ-DI.

Supplementary Figure 8


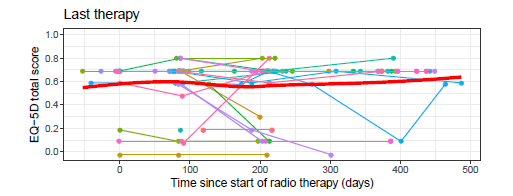


Supplementary Figure 8a


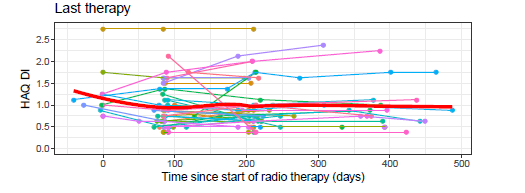


Supplementary Figure 8

Supplementary Figure 9.

Plantar fasciitis: there seems to be a slight increase in EQ-5D total score while no such pattern can be seen for the HAQ-DI.

Supplementary Figure 9


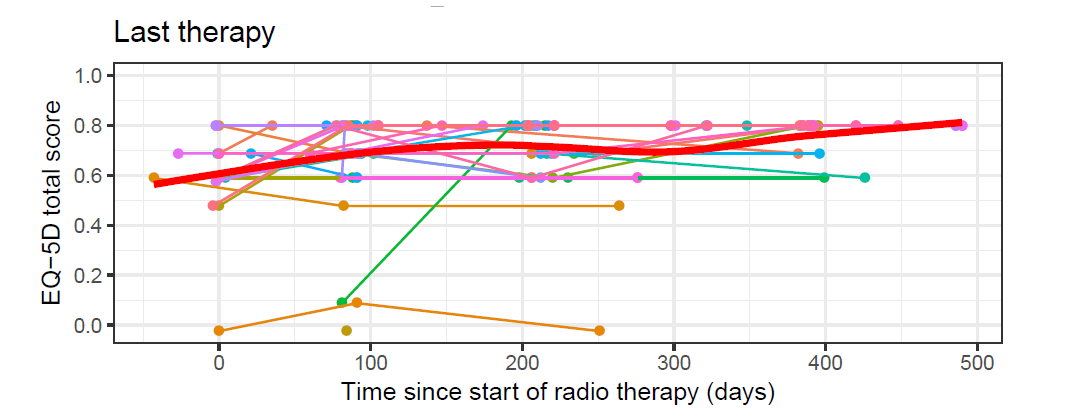


Supplementary Figure 9a


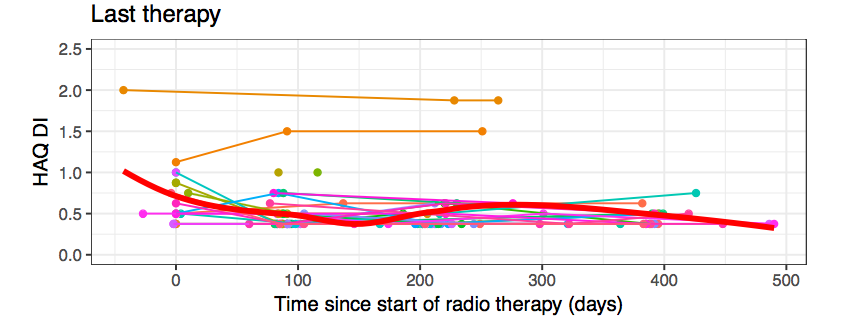


Supplementary Figure 9b

**Supplementary Tables**

**Supplementary Table 1**

Lateral epicondylitis: Summary statistics table for outcomes after last LDRT. *n* refers to the number of treated sites.

**Supplementary Table 1**

| Variable | Levels | n | x̃ | s | x̅ | q1 | q3 | Min | Max |
| --- | --- | --- | --- | --- | --- | --- | --- | --- | --- |
| Handgrip strength (kg) elbow flexion | 0 | 38 | 25.9 | 11 | 24.5 | 17.2 | 32.8 | 8 | 51 |
|  | 2 | 38 | 25.7 | 11.4 | 26 | 17.5 | 32.5 | 5 | 51 |
|  | 6 | 33 | 28.3 | 12.5 | 28 | 20 | 38 | 0 | 49 |
|  | 12 | 30 | 29.2 | 9.7 | 26.5 | 23 | 33 | 13.3 | 48.5 |
|  | all | 139 | 27.1 | 11.2 | 26 | 19.8 | 34 | 0 | 51 |
| Handgrip strength (kg)  elbow extension | 0 | 38 | 21.5 | 13.1 | 19.5 | 9.8 | 27.8 | 0.1 | 45 |
|  | 2 | 38 | 24.1 | 12.8 | 23 | 15 | 31.5 | 0 | 49 |
|  | 6 | 33 | 28.2 | 13.3 | 27 | 20 | 41 | 0 | 50 |
|  | 12 | 30 | 29.1 | 9.9 | 26 | 22.6 | 35 | 11 | 50 |
|  | all | 139 | 25.4 | 12.7 | 24 | 17 | 35 | 0 | 50 |
| Pain (VAS)  at rest | 0 | 37 | 2.9 | 2.9 | 3 | 0 | 5 | 0 | 9 |
|  | 2 | 38 | 1.4 | 2.2 | 0 | 0 | 3 | 0 | 8 |
|  | 6 | 33 | 1.6 | 2.5 | 0 | 0 | 2.5 | 0 | 8 |
|  | 12 | 31 | 1 | 2 | 0 | 0 | 1 | 0 | 8 |
|  | all | 139 | 1.8 | 2.5 | 0 | 0 | 3 | 0 | 9 |
| Pain (VAS)  during activity | 0 | 37 | 5.7 | 2.6 | 6 | 4 | 7 | 0 | 10 |
|  | 2 | 38 | 3.5 | 2.7 | 3 | 1.1 | 5 | 0 | 10 |
|  | 6 | 33 | 2.7 | 2.8 | 2 | 0 | 4 | 0 | 10 |
|  | 12 | 30 | 2.3 | 2.9 | 1 | 0 | 3 | 0 | 9 |
|  | all | 138 | 3.6 | 3 | 3 | 1 | 6 | 0 | 10 |

**Supplementary Table 2**

Medial epicondylitis: Summary statistics table for outcomes after last LDRT. *n* refers to the number of treated sites.

**Supplementary Table 2**

| Variable | Levels | n | x̃ | s | x̅ | q1 | q3 | Min | Max |
| --- | --- | --- | --- | --- | --- | --- | --- | --- | --- |
| Handgrip strength (kg)  elbow flexion | 0 | 10 | 27 | 12.3 | 26 | 17.2 | 33 | 13 | 52 |
|  | 2 | 9 | 23.7 | 12.8 | 22 | 11 | 29 | 10 | 46 |
|  | 6 | 7 | 34.8 | 14.9 | 34 | 25.5 | 42.8 | 15 | 58 |
|  | 12 | 7 | 36 | 13.1 | 35 | 28.5 | 42.2 | 18.5 | 57 |
|  | all | 33 | 29.7 | 13.6 | 29 | 18.5 | 39 | 10 | 58 |
| Pain (VAS)  at rest | 0 | 10 | 3.2 | 2.6 | 3.5 | 1 | 5.8 | 0 | 6 |
|  | 2 | 8 | 3.2 | 3.6 | 1.8 | 0 | 6.5 | 0 | 8 |
|  | 6 | 7 | 0.8 | 1.9 | 0 | 0 | 0.2 | 0 | 5 |
|  | 12 | 6 | 0.8 | 2 | 0 | 0 | 0 | 0 | 5 |
|  | all | 31 | 2.2 | 2.8 | 0.5 | 0 | 5 | 0 | 8 |
| Pain (VAS)  during activity | 0 | 10 | 4.9 | 2.8 | 6 | 3.2 | 6.8 | 0 | 9 |
|  | 2 | 8 | 3.9 | 3.4 | 4.5 | 0.4 | 6.5 | 0 | 8 |
|  | 6 | 7 | 2.4 | 3.8 | 0.5 | 0 | 3 | 0 | 10 |
|  | 12 | 6 | 1.3 | 2.2 | 0 | 0 | 2.2 | 0 | 5 |
|  | all | 31 | 3.4 | 3.3 | 3 | 0 | 6 | 0 | 10 |

**Supplementary Table 3**

Finger osteoarthritis: Summary statistics table for outcomes after last LDRT. *n* refers to the number of treated sites.

**Supplementary Table 3**

| Variable | Time point | n | x̃ | s | x̅ | q1 | q3 | Min | Max |
| --- | --- | --- | --- | --- | --- | --- | --- | --- | --- |
| Handgrip strength (kg) | 0 | 100 | 17.3 | 8 | 16.5 | 11.8 | 23.2 | 2 | 42 |
| elbow flexion | 2 | 98 | 17.8 | 7.9 | 18 | 12.2 | 22.4 | 0 | 37.5 |
|  | 6 | 85 | 18.1 | 8.7 | 18 | 12 | 22 | 0 | 46 |
|  | 12 | 82 | 18.2 | 8.7 | 18.2 | 14 | 22 | 0 | 43 |
|  | all | 365 | 17.8 | 8.3 | 18 | 12 | 22.5 | 0 | 46 |
| Pinch grip strength (kg) | 0 | 65 | 5.2 | 2 | 5 | 4 | 6.5 | 1 | 12.8 |
|  | 2 | 64 | 5.2 | 2.2 | 5.4 | 4.4 | 6 | 0 | 12.5 |
|  | 6 | 53 | 5.4 | 2.1 | 5.5 | 4.5 | 6.5 | 0 | 12 |
|  | 12 | 53 | 4.8 | 2 | 5 | 3.5 | 6 | 2 | 12 |
|  | all | 235 | 5.2 | 2.1 | 5 | 4 | 6 | 0 | 12.8 |
| Pain (VAS)  at rest | 0 | 100 | 2.6 | 2.4 | 2 | 0 | 4 | 0 | 10 |
|  | 2 | 99 | 2.3 | 2.6 | 1.5 | 0 | 4 | 0 | 10 |
|  | 6 | 86 | 1.9 | 2.5 | 0 | 0 | 3 | 0 | 9 |
|  | 12 | 83 | 2.4 | 3 | 0.5 | 0 | 5 | 0 | 10 |
|  | all | 368 | 2.3 | 2.6 | 1 | 0 | 4 | 0 | 10 |
| Pain (VAS)  during activity | 0 | 100 | 5.4 | 2.4 | 5 | 4 | 7.1 | 0 | 10 |
|  | 2 | 99 | 4.7 | 2.8 | 4 | 2.5 | 7 | 0 | 10 |
|  | 6 | 86 | 3.9 | 2.9 | 4 | 1 | 6 | 0 | 10 |
|  | 12 | 83 | 4.1 | 3.1 | 4 | 1 | 7 | 0 | 10 |
|  | all | 368 | 4.6 | 2.8 | 5 | 2 | 7 | 0 | 10 |
|  |  |  |  |  |  |  |  |  |  |

**Supplementary Table 4**

Plantar fasciitis: Summary statistics table for outcomes after last LDRT. *n* refers to the number of treated sites.

**Supplementary Table 4**

| Variable | Time point | n | x̃ | s | x̅ | q1 | q3 | Min | Max |
| --- | --- | --- | --- | --- | --- | --- | --- | --- | --- |
| Walking test (seconds) | 0 | 56 | 27.6 | 9.4 | 25.5 | 23.0 | 29.0 | 14 | 72 |
|  | 2 | 50 | 25.3 | 7.8 | 24.0 | 20.2 | 26.8 | 12 | 60 |
|  | 6 | 43 | 24.3 | 10.1 | 22.0 | 20.0 | 26.0 | 10 | 74 |
|  | 12 | 39 | 23.2 | 7.7 | 22.0 | 20.0 | 25.0 | 11 | 60 |
|  | all | 188 | 25.3 | 8.9 | 23.0 | 20.0 | 27.0 | 10 | 74 |
| Pain (VAS)  at rest | 0 | 55 | 3.1 | 3.3 | 1.0 | 00 | 6.0 | 0 | 10 |
|  | 2 | 53 | 1.3 | 2.7 | 0.0 | 0.0 | 1.0 | 0 | 10 |
|  | 6 | 44 | 0.8 | 2.2 | 0.0 | 0.0 | 0.0 | 0 | 10 |
|  | 12 | 41 | 0.4 | 1.5 | 0.0 | 0.0 | 0.0 | 0 | 7 |
|  | all | 193 | 1.5 | 2.8 | 0.0 | 0.0 | 1.0 | 0 | 10 |
| Pain (VAS) during activity | 0 | 55 | 5.6 | 2.6 | 6.0 | 3.8 | 8.0 | 1 | 10 |
|  | 2 | 52 | 2.5 | 2.8 | 1.5 | 0.0 | 4.2 | 0 | 10 |
|  | 6 | 44 | 1.2 | 2.4 | 0.0 | 0.0 | 1.0 | 0 | 10 |
|  | 12 | 41 | 1.0 | 1.9 | 0.0 | 0.0 | 1.0 | 0 | 7 |
|  | all | 192 | 2.8 | 3.1 | 1.0 | 0.0 | 5.0 | 0 | 10 |
